# Magnetic cobalt metal organic framework for photocatalytic water splitting hydrogen evolution

**DOI:** 10.1186/s11671-024-04019-3

**Published:** 2024-05-07

**Authors:** Mohammad Hossein Razeghi, Ozra Gholipour, Jaber J. Sardroodi, Sajjad Keshipour, Ali Hassanzadeh

**Affiliations:** 1 Department of Chemistry, Azarbaijan Shahid Madani University, Tabriz, Iran; 2 Department of Nanotechnology, Faculty of Chemistry, Urmia University, Urmia, Iran; 3 Department of Physical Chemistry, Faculty of Chemistry, Urmia University, Urmia, Iran

**Keywords:** Nanostructure, Hydrogen generation, Metal–organic frameworks, Magnetic nano particles, Green chemistry

## Abstract

Using water as a renewable and safe energy source for hydrogen generation has reduced the need to use toxic fossil fuels. Photocatalytic approaches provide a worthy solution to avoid the high expenditure on complicated electrochemical pathways to promote Hydrogen Evolution Reactions. However, several types of photocatalysts including noble metal-based catalysts have already been in use for this purpose, which are generally considered high-cost as well. The present study aims to use the benefits of metal–organic frameworks (MOFs) with semiconductor-like characteristics, highly porous structures and high design flexibility. These properties of MOFs allow more efficient and effective mass transport as well as exposure to light.in this paper, using MOF technology and benefiting from the characteristics of Fe_3_O_4_ nanoparticles as catalyst support for more efficient separation of catalyst, we have synthesized a novel composite. Our proposed photocatalyst demonstrates efficient harvest of light in all wavelengths from UV to visible to generate electron/hole pairs suitable for water splitting with a turnover frequency of 0.222 h^−1^ at ambient conditions without requiring any additives.

**Graphical abstract:**

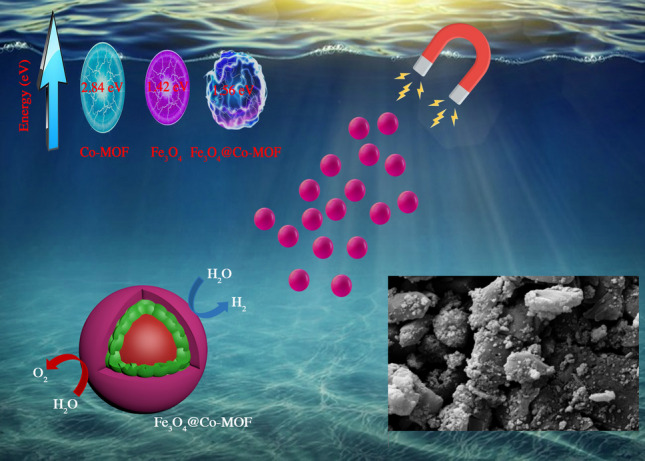

## Introduction

As the world’s population is growing and industrialization accelerates, the use of non-sustainable fossil fuels has triggered a global energy crisis and raised significant environmental concerns, such as greenhouse gases, water contamination, and air pollution [1]. Consequently, developing sustainable alternative and clean energy is an effective strategy to reduce our high dependency on fossil fuels in the twenty-first century; ergo, hydrogen energy is considered a promising alternative energy source based on its extensive sources, and high gravimetric energy density [2, 3]. In comparison to gasoline, hydrogen fuel is less polluting, and toxic with a higher capacity for heat and power production, and combustion efficiency. Generation of hydrogen through photocatalytic water splitting has attracted considerable attention recently mainly owing to its potential applications in renewable and clean energy production [4, 5]. A variety of nanocomposite photocatalysts have demonstrated excellent photocatalytical activity in recent years as a result of their unique structural composition, and electronic charge transfer capabilities [6, 7]. Hydrogen fuels can be produced by a wide range of inorganic semiconductor photocatalysts, such as TiO_2_ [8], CdS [9], BiVO_4_ [10], NaTaO_3_ [11], and MIL-100(Fe) [12]. Nevertheless, current materials have low conversion efficiency due to their inadequate light absorption capacity, narrow photoresponse, rapid recombination, and poor photostability. Therefore, the development of more efficient photocatalysts as precious metal substitutes is urgently necessary [13].

The metal–organic frameworks (MOFs) have recently been considered promising candidates for several fields, including gas sorption and separation, catalysis, and sensing, as a result of their stability chemical structure, large surface area, size and shape adaptability, and high porosity [14–16]. In contrast to conventional photocatalysts, MOFs characterized by semiconductor-like characteristics exhibit a highly porous structure, design flexibility, and the ability to expose catalytic sites during photocatalytic reactions, which facilitates mass transport of reactants and products [17, 18]. Different types of MOFs have been reported to be useful for photocatalysis of hydrogen evolution, including UiO-66(Zr) [18], MOF/MoS_2_ [7], Ni-based MOF [19], and Fe-based MOF [12], as a result of their unique characteristics including thermal stability, large surface area, high porosity (90% free volume), dynamic structure, and crystallization. Cobalt (Co)-based MOFs are considered viable catalysts due to their cost-effectiveness, high efficiency, and stability, ease of preparation, and environmental protection properties. Photocatalytic activity can also be enhanced by adjusting the specific surface area and band gap of MOFs [20].

Water splitting is considered as one of the significant strategies to produce hydrogen using a free and abundant source [21]. Two main pathways are employed to split water, including electrolysis by instruments, and photolysis by photocatalysts. The photocatalytic procedure stands out due to the benefits like inexpensive nature, easy handling, high efficiency, and long durability. Some important photocatalysts have been introduced in the recent years such as Ni-MOF-74/Ni_2_P/MoSx, CdS/Ni-MOF, and Ni-MOF–74/BiVO_4_ [22–24]. Although most of the reports afforded high yields, two main challenges have not been responded, including easy synthesis, and recovery. To solve the problem, herein, we used Co-MOF as an economical photocatalyst, and magnetic nanoparticles to magnetize the photocatalyst in order to achieve a facile final separation stage the final easy separation from the reaction mixture. Therefore, terephthalic acid was used as the raw material in the synthesis of MOF in the presence of Fe_3_O_4_ nanoparticles. Magnetic nanoparticles have been demonstrated to be reliable heterogeneous catalysts and catalyst supports in comparison to conventional materials [25, 26]. In addition to the simplicity of the synthesis procedure, Fe_3_O_4_, as the nucleation seeds, leads to the formation of fine particles of MOF via preventing aggregation of MOF particles. The obtained magnetically recoverable photocatalyst could promote the water splitting reaction in high yield. To compare the obtained results with the previous reports, hydrogen production rate is mentioned as Turnover Frequency (TOF), which is defined as the mol of generated hydrogen per the mol of catalyst per unit time. A higher TOF means a more efficient catalyst [27, 28].

## Experimental

### Materials

All reagents were used without further purification and were of the highest purity. Materials for the experiment were obtained from Merck and Sigma-Aldrich, including Co (NO_3_)_2_.6H_2_O, N, N-Dimethylformamide (DMF)(C_3_H_7_NO), Terephthalic acid (C_8_H_6_O_4_), FeCl_2_.4H_2_O, FeCl_3_.6H_2_O, Ammonia solution (NH_3_) and ethanol. Throughout the procedure, deionized water (DI) was used to prepare all solutions and suspensions.

### Synthesis of Fe_3_O_4_

In this study, Fe_3_O_4_ nanoparticles were synthesized using a facile co-precipitation technique. FeCl_2_ 0.4H_2_O and FeCl_3_. 6H_2_O with a molar ratio 2:1 was dissolved in distilled water (30ml) under mechanical stirring at room temperature. Afterwards, drop by drop, ammonia solution (25%, 1.5 ml) under a nitrogen atmosphere was added to the mixture over a period of 20 min at 80 °C. After obtaining a black solution, it was stirred for one hour at 80 °C under a nitrogen atmosphere. The resultant precipitate was collected by an external magnet and thoroughly washed with deionized water and dried at 70 °C under vacuum.

### Synthesis of Fe_3_O_4_@Co-MOF

The preparation of Fe_3_O_4_@Co-MOF was straightforward. To synthesize Fe_3_O_4_@Co-MOF, Fe_3_O_4_ nanoparticles in the desired proportion were dispersed in 15 ml of DMF to produce stable Fe_3_O_4_ nanoparticles. In the following steps, terephthalic acid (0.453 gr) was added to the reaction mixture under ultrasound for a period of 30 min. The next step consisted of adding drop by drop Co (NO_3_)_2_.6H_2_O solution to the obtained solution of Fe_3_O_4_/terephthalic acid in the ultrasonic cleaner for 30 min. After ultra-sonication for 30 min at room temperature, the product was transferred to 100 ml of a Teflon-lined stainless-steel autoclave heated at 80 °C for 24 h in an oven. In order to collect the resulting magnetic catalyst, an external magnetic force was applied and the catalyst was repeatedly washed with DMF and ethanol 96%. For 12 h, the final product was dried in a vacuum oven at 60 °C.

### Typical procedure for water splitting

To perform photocatalytic water splitting, a 200 W xenon lamp with a wavelength of > 420 nm was used in conjunction with 10 mL of distilled water and 0.01 gr of Fe_3_O_4_@Co-MOF in a vessel that was connected to an inverted burette filled with water in order to capture the generated gas. A dark room was initially used for the mixing of the reaction mixture for 10 min, and then light was radiated onto it. Finally, to calculate the volume of hydrogen, the volume changes in the burette was recorded every 10 min. Based on the assumption that both H_2_ and O_2_ function as ideal gases, the volume of H_2_ was calculated. The following equation was used to calculate TOF within 10 min:

$${\text{TOF}}\left( {{\text{h}}^{{ - {1}}} } \right) = \left( {{\text{PV}}} \right)/\left( {{\text{2R}}.{\text{T}}.{\text{ n}}.{\text{t}}} \right)$$

In this equation, *P* is atmospheric pressure (atm), V is the volume of gas produced (ml), R is the universal gas constant (0.08206 L. atm/mol. K), T is temperature (K), n is the amount of Co present in the catalyst used, and t is time (h).

**Fig. 1:**
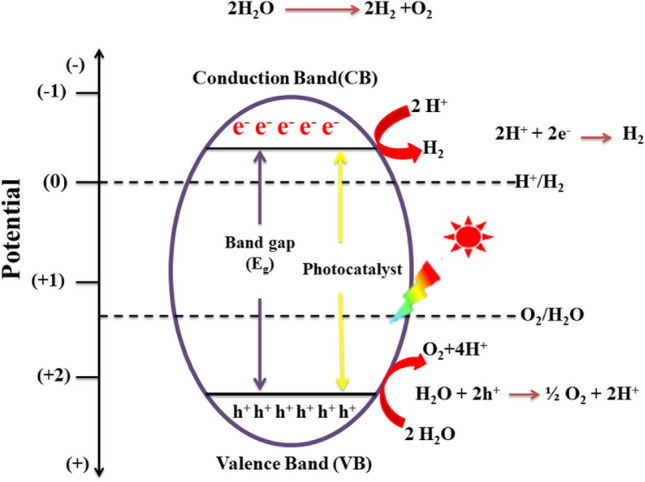
Mechanism of the photocatalytic water splitting

A proposed mechanism of photocatalytic hydrogen production is depicted in Fig. 1. The mechanism includes light absorption, electron–hole formation, and generation of H_2_/O_2_. Photocatalyst absorb light’s energy in the form of ultraviolet, visible, or infrared light. The excited electrons move from the valence band to the conduction band, producing electron–hole pairs. The injected electrons generate hydrogen from protons, and the remained holes push the O_2_ generation. The role of semiconductors in the acceleration of this phenomenon is extremely important, and comprehensive research is required to develop efficient nanocomposites as the semiconductors [29, 30].

### Sample characterization

A X’Pert Pro instrument with Cu K1 radiation at a wavelength of 1.54 Å was used to measure the X-ray Diffraction (XRD) pattern of the as-prepared samples. Fourier-transform infrared spectroscopy (FTIR) of the prepared materials in the range 400–4000 cm^−1^ was conducted with Spectrum Two from PerkinElmer with a standard KBr beam splitter. Field Emission Scanning Electron Microscopes (FE-SEM, model SIGMA VP) were used to examine the morphological characteristics of the prepared sample. With the Oxford instrument, energy-dispersive X-ray (EDS) spectroscopy and mapping images were collected. A TEM was used to examine the samples (Philips BioTwin, Netherlands). Measurement of magnetic properties was carried out using a vibrating sample magnetometer (VSM-model LBKFB, Meghnatis Daghigh Kavir Company). A BET method was used to determine the surface area. In order to determine the size and volume of the catalyst pores, a multiple point N_2_ adsorption–desorption method based on the Barrett-Joyner-Halenda (BJH) model was employed.

## Results and discussion

### FT-IR

**Fig. 2:**
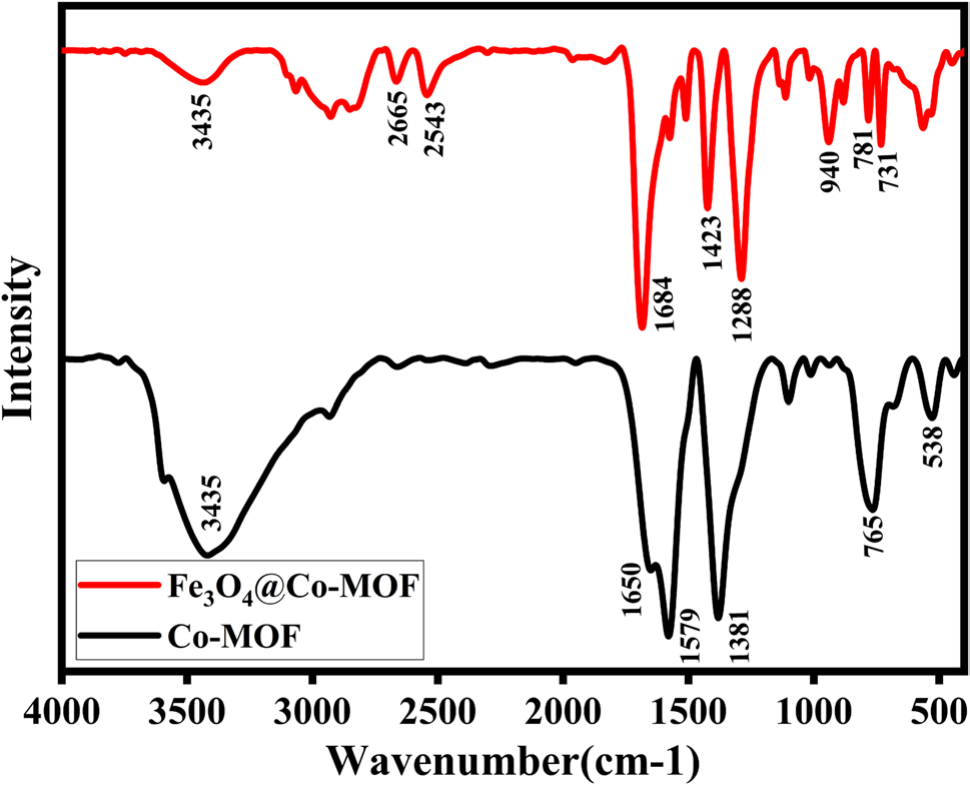
FT-IR spectra of Fe_3_O_4_@Co-MOF and Co-MOF

Figure 2 displays the FT-IR spectra of Fe_3_O_4_@Co-MOF, and Co-MOF, respectively, confirming the existence of functional groups in these catalysts. As shown in the FT-IR spectrum of CO-MOF and Fe_3_O_4_, the intensity and position of the peaks are identical to those reported in the literature [2, 31, 32]. Organic linker BDCs contain main functional groups corresponding to C=O bonds in the –COO– groups located in the wavelength range between 1800 and 1400 cm^−1^. The peak at 1380 cm^−1^ corresponds to the stretching vibration of the C–O bond, and the peaks at 1570 and 1662 cm^−1^ indicate the presence of a C=C bond and a C=O stretching vibration from the carbonyl C=O of 1,4-benzenedicarboxylic acid. In addition, the absorption peak at approximately 538 cm^−1^ is attributed to Co–O, which decreases in intensity as Co (NO_3_)_2_.6H_2_O interacts with 1,4-benzenedicarboxylic acid. As well, the peak at 765 cm^−1^ in the Co-MOF spectra is related to the stretching vibrations of the C–H bond in the benzene ring. The presence of hydroxyl groups can be attributed to a broad band at 3435 cm^−1^. As a result of the reaction between Fe_3_O_4_ and MOF, the spectra of Fe_3_O_4_@Co-MOF indicate peaks that shift all bonds. For Fe_3_O_4_@Co-MOF composite samples, those that are ascribed to MOF are still evident on the FT-IR spectra and several peaks that are attributed to Fe_3_O_4_ NPs are overlapped; however, the absorption peaks at 564 cm^−1^ and 731 cm^−1^ belonged to the stretching vibration mode of Fe–O from the magnetic nanoparticle cores. The characteristic peaks appear between 2900 and 2500 cm^−1^ due to C–H stretching. Ergo, it can be confirmed that magnetized Fe_3_O_4_@Co-MOF has been successfully synthesized.

### XRD

**Fig. 3:**
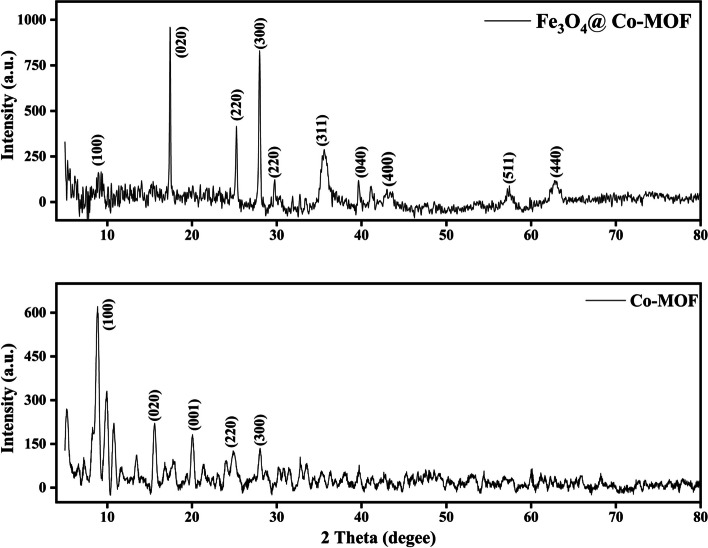
X-ray diffraction (XRD) of Fe_3_O_4_@Co-MOF and Co-MOF

XRD was used to evaluate the crystal structure of the catalyst. Nanoparticle crystallinity and average nanoparticle diameter can both be determined using XRD. Figure 3 illustrates the XRD patterns for CO-MOF and Fe_3_O_4_@Co-MOF composite samples [2, 31, 32]. Diffraction peaks of CO-MOF are 2theta = 10°, 15°, 25°, and 30°, which correspond to its crystal planes (100), (020), (001), and (300), as reported in the literature [33, 34]. Furthermore, the additional characteristic peaks associated with planes (220), (311), (400), (511) and (440) in the Fe_3_O_4_@Co-MOF pattern, located at around 30°, 35°, 43°, 57°, and 62°, are consistent with the crystalline structure of Fe_3_O_4_, which is in accordance with existing literature [2, 35]. In comparison with Co-MOF particles, Fe_3_O_4_@Co-MOF particles exhibit a similar XRD pattern, indicating that the crystal structure of Co-MOF was not transformed after Fe_3_O_4_ particles were prepared.

### FESEM and TEM

**Fig. 4:**
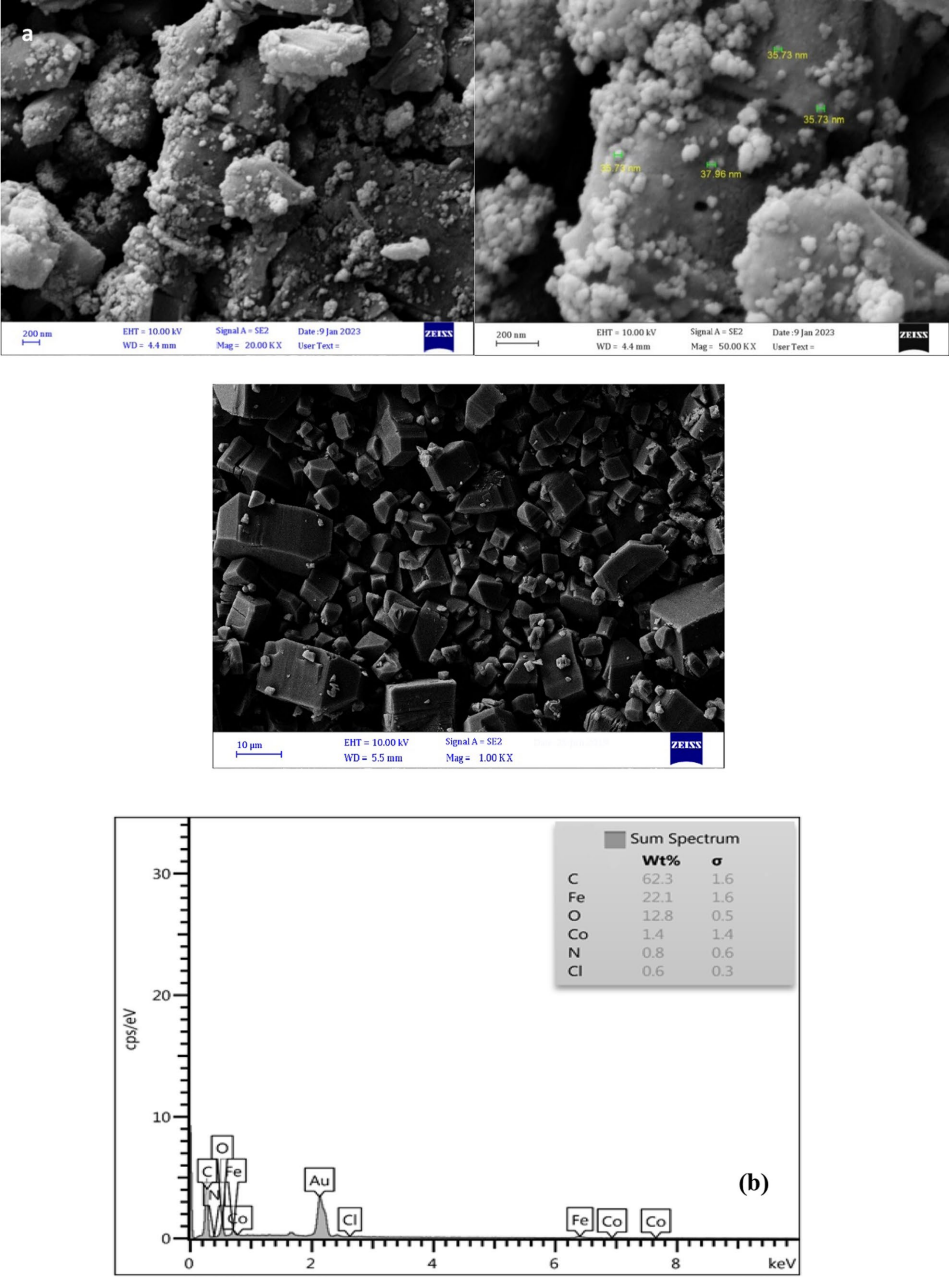
FESEM image of Fe_3_O_4_@ Co-MOF and Co-MOF (**a**), and EDX analysis of Fe_3_O_4_@Co-MOF (**b**)

**Fig. 5:**
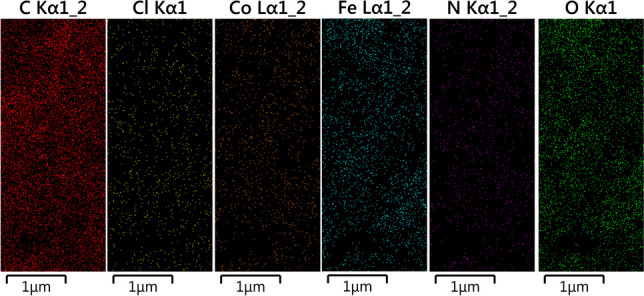
Elemental mapping of Fe_3_O_4_@Co-MOF

**Fig. 6:**
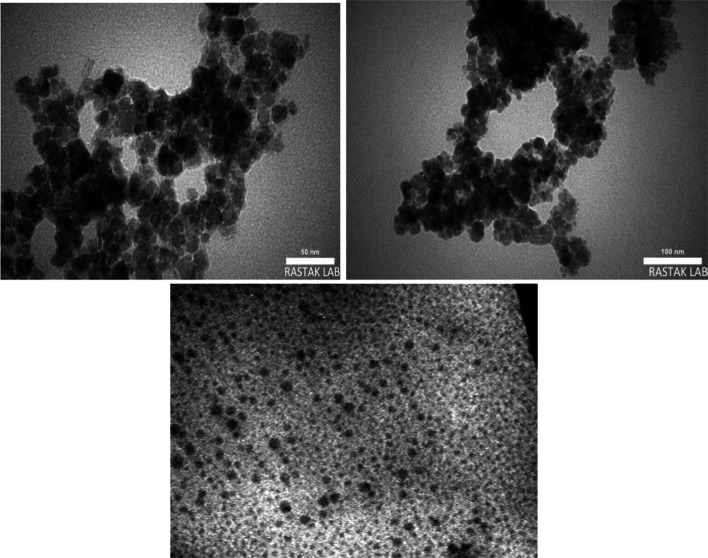
TEM images of Fe_3_O_4_@Co-MOF and Co-MOF

FESEM and TEM have been used to clarify the surface morphology, size, and microstructure of the as-prepared magnetic Fe_3_O_4_@Co-MOF and Co-MOF. As can be seen in Fig. 4a, the Co-MOF particle displays an irregular crystal structure, and the majority of the Fe_3_O_4_ nanoparticles with their spherical shape are directly attached to the surface of the MOF without damaging its structure. Magnetic Fe_3_O_4_ nanoparticles have a diameter of approximately 35 nm. The results of the EDX spectroscopy analysis of the corresponding nanoparticles (Fig. 4b) confirm the presence and uniform distribution of the expected elements, including C, N, O, Fe, Cl, and Co. According to the quantitative analysis of EDX results, the proportion of each element in the sample was determined. as seen in the mentioned figure Carbon, which accounts for 62.3% of the catalyst, has a higher proportion than other elements namely Iron (22.1%), Oxygen (12.8%), Cobalt (1.4%), Nitrogen (0.8%), and Chloride (0.6%). In addition, elemental mapping was employed to verify the uniform distribution of Co, Fe, N, C, Cl, and O elements throughout the sample, as well as to verify the formation of catalysts (Fig. 5). TEM analysis provides additional information regarding the morphology and state of as-synthesized Fe_3_O_4_@Co-MOF magnetic NPs. The TEM image of the Fe_3_O_4_@Co-MOF and Co-MOF composite sample is shown in Fig. 6. Due to the small particle size of Fe_3_O_4_ core, the TEM image of Fe_3_O_4_@Co-MOF magnetic NPs reveal agglomerated Fe_3_O_4_ NPs within the core–shell structure. The TEM image of Fe_3_O_4_@Co-MOF and Co-MOF in the Fig. 6 demonstrated clearly that Fe_3_O_4_ nanoparticles were attached to the surface of MOF.

### BET analysis

**Fig. 7:**
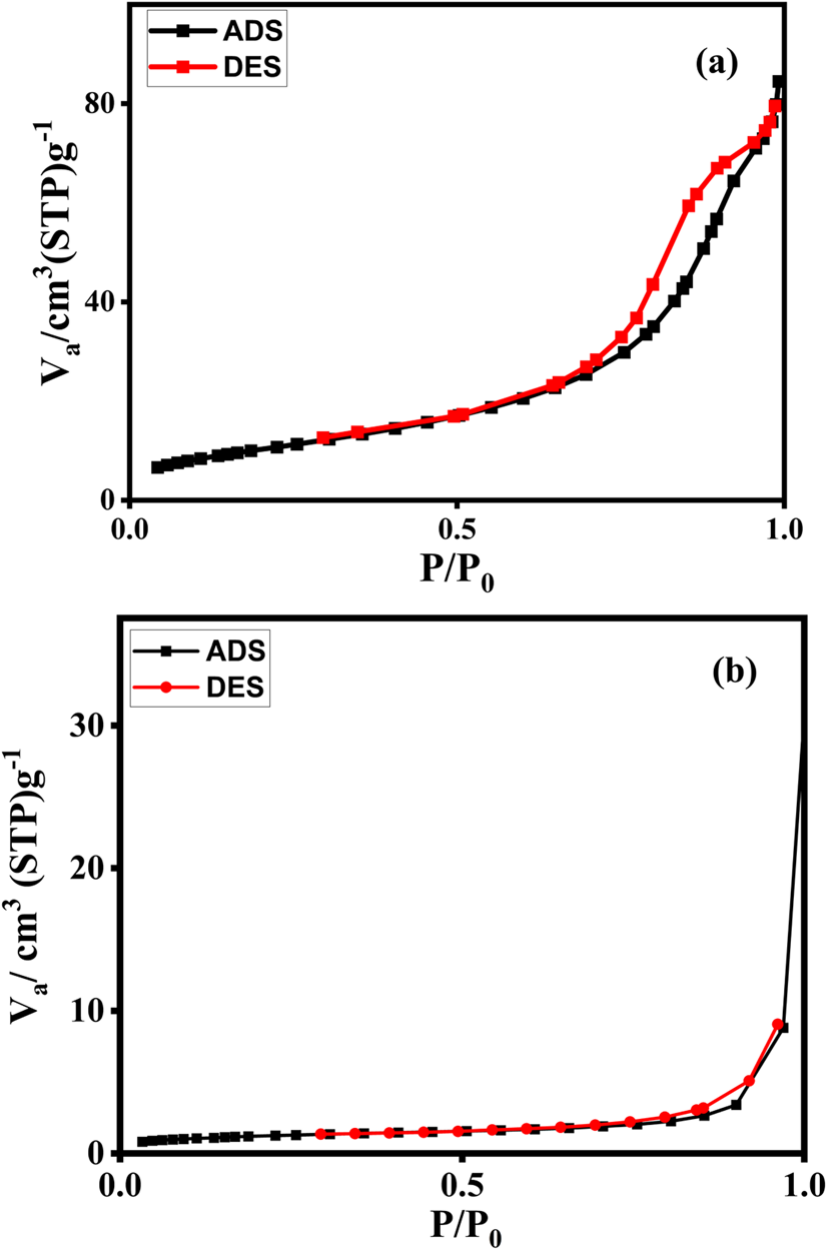
N2 adsorption–desorption isotherm of (**a**) Fe_3_O_4_@Co-MOF and (**b**) Co-MOF

As can be seen in Fig. 7a, b, the N_2_ adsorption–desorption isotherm of Co-MOF and Fe_3_O_4_@Co-MOF was used as a measure of the specific surface area and porosity of the surface within the catalyst system. According to the IUPAC classification system, the isotherm of Fe_3_O_4_@Co-MOF is similar to a type III isotherm, which implies that the samples are mesopores with obvious hysteresis loops, possibly due to capillary condensation in the pores. The isotherm of Co-MOF is similar to the type III isotherm, indicating that Co-MOF possesses mesoporous and macroporous characteristics. Details of the isotherm studies are provided in the Table 1. An adsorbent's total surface area is determined by the BET isotherm (including its outer surface and pore surface). In contrast, the t-plot is used to measure the outer surface area (external surface area). The internal surface area is determined by the difference between the BET surface area and the t-plot. We used the Barret-Joyner-Halenda (BJH) method to examine the pore size distribution (PSD). According to the results, the surface properties of Fe_3_O_4_@Co-MOF is summarized as S_BET_ = 39.45 m^2^g^−1^, V_m_ = 9.06 cm^3^g^−1^, the average pore diameter of Fe_3_O_4_@Co-MOF is 12.93 nm, and the total volume of the pore is 0.127 cm^3^g^−1^. It is notable that the larger pores provide more available active adsorption sites. As a result, the majority of the surface reported in the BET method is the external surface, as presented in Table 1.

**Table 1:** The BET textural properties of Fe_3_O_4_@Co-MOF

Material name	BET		t-plot	BJH
	BET surface area (cm^2^g^−1^)	Pore volume (cm^3^g^−1^)	External surface area (m^2^g^−1^)	Rpore (nm)
Fe_3_O_4_@Co-MOF	39.45	0.1276	34.11	5.35
Co-MOF	4.37	1.0	2.42	12.52

### UV and band gap and CV

**Fig. 8:**
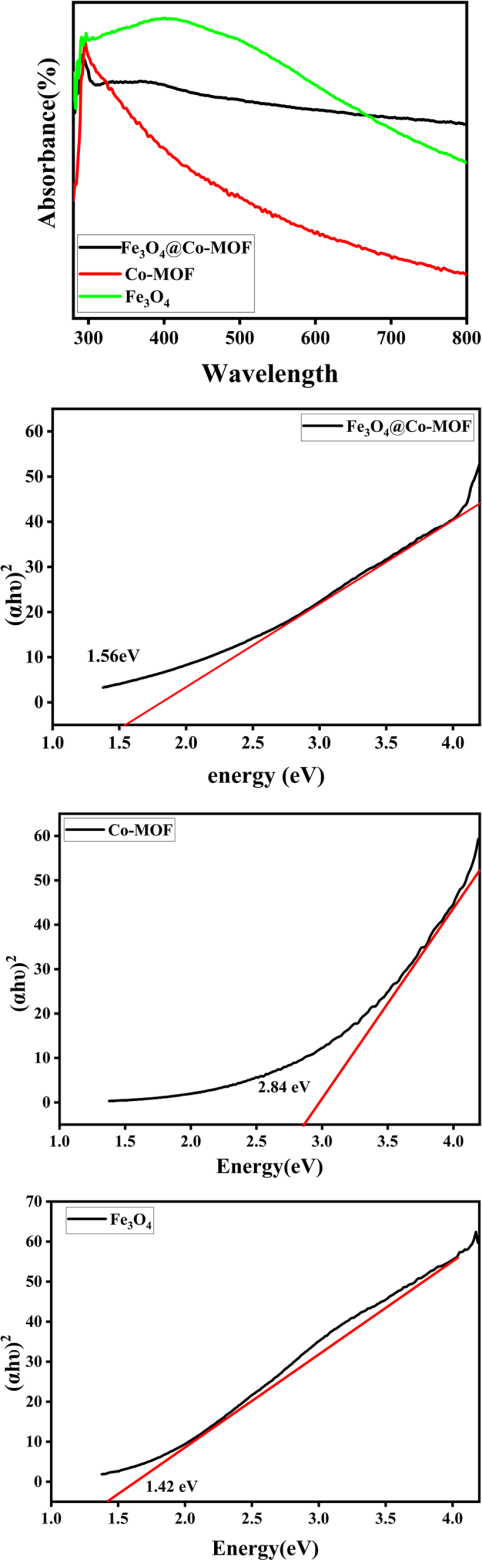
UV–VIS spectrum, Tauc plot of Fe_3_O_4_@Co-MOF, Fe_3_O_4_, and Co-MOF

**Fig. 9:**
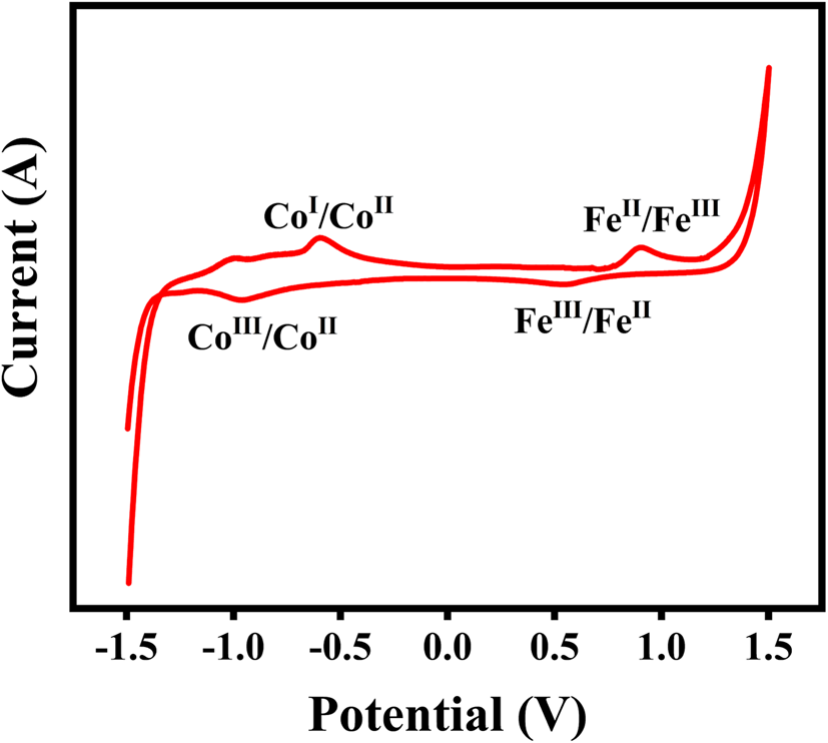
CV diagram of Fe_3_O_4_@Co-MOF

A UV–Vis spectrum of Co-MOF, before and after the deposition of Fe_3_O_4_ nanoparticles, was provided prior to starting the photocatalytic reaction using magnetic metal organic framework (Fig. 8a). This was mainly performed in order to compare their optical absorption properties and to determine the effect of Fe_3_O_4_ loading on MOF on its absorbance properties. Therefore, a stock solution was prepared by dissolving a definite portion of catalysts in distilled water and the water solution was used as a blank sample to calibrate. Among the most powerful photocatalysts, those with broad absorption in the visible region are of particular interest [21]. There was absorbance in a wide range of UV and Vis regions across all spectra, indicating that Fe_3_O_4_@Co-MOF can be operated over a wide range of UV–Vis. It was suggested that this fascinating absorption could have facilitated a photocatalytic water-splitting reaction. The observed redshifts were attributed to the loading of Fe_3_O_4_ in comparison to bare Fe_3_O_4_ and Co-MOFs which were reported at about 298, and 293 nm, respectively. The bandgap Tauc plots of Fe_3_O_4_, Co-MOF and Fe_3_O_4_@Co-MOF were provided (Fig. 8b–d), obtaining values of 1.42, 2.84, and 1.56 eV, respectively. As a result of loading Fe_3_O_4_ onto Co-MOF, substantial enhancements in its optical activity were observed. As a result of the modification reaction toward Co-MOF synthesis, the band gap was decreased to 1.56 eV, creating an excellent opportunity for harvesting light in the visible region, a safe zone for UV–vis photos, and an increase in absorbance efficiency. Since electron transfer occurs from highest occupied molecular orbital (HOMO) to the lowest unoccupied molecular orbital (LUMO), cyclic voltammetry (CV) was carried out to determine the energy levels according to Leeuw’s formula (E_HOMO_ =—(oxidation potential (E_ox_) + 4.71) eV) [13, 36]. The CV plot of Fe_3_O_4_@Co-MOF in the range of – 2–2 V gave the value of E_HOMO_ as − 4.124 eV (Fig. 9). Considering the band gap of 2.84 eV, the value of E_LUMO_ was calculated as − 1.284 eV.

**Fig. 10:**
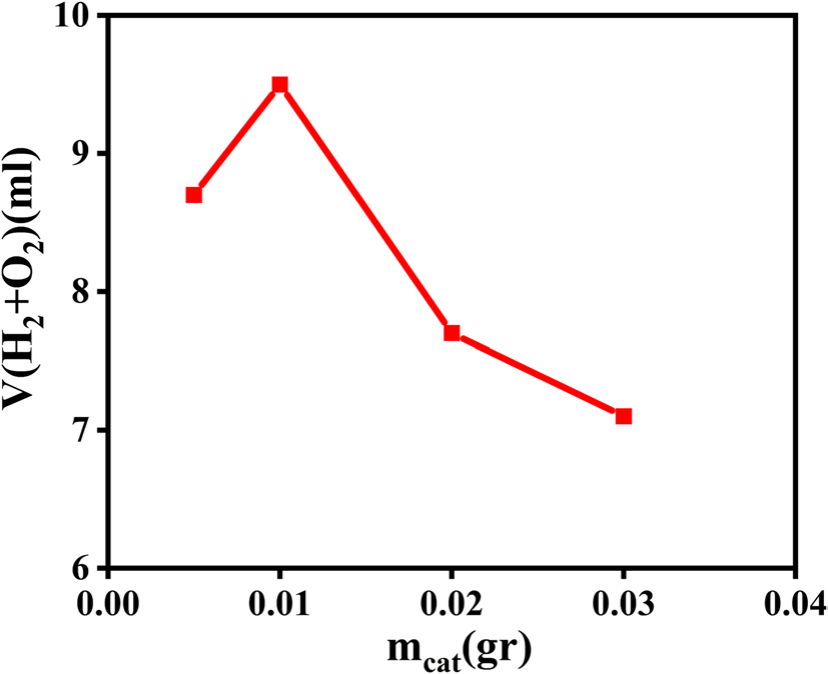
Effects of Fe_3_O_4_@Co-MOF amounts

An important parameter in all photocatalytic reactions is the amount of photocatalyst used. In the range of 5–30 mg, the effect of magnetic Co-MOF on photocatalytic hydrogen evolution was examined. The hydrogen yield, with respect to varying doses, is shown in Fig. 10. The photocatalytic activity increased initially until reaching 10 mg, and then decreased with higher doses. With a lower dose, the number of active sites is also lower, resulting in a lower evolution of hydrogen. With increasing doses, hydrogen evolution initially increases; However, if the dose continues to rise, turbidity will impede further penetration of light into the reactor after travelling a certain distance. This indicates that light penetration has been limited, which leads to a decrease in hydrogen yield. For the most efficient hydrogen production, the effective dose of photocatalyst was found to be 10 mg of catalyst in 10 min under 200 W irradiation with a TOF of 0.222 h^−1^.

**Fig. 11:**
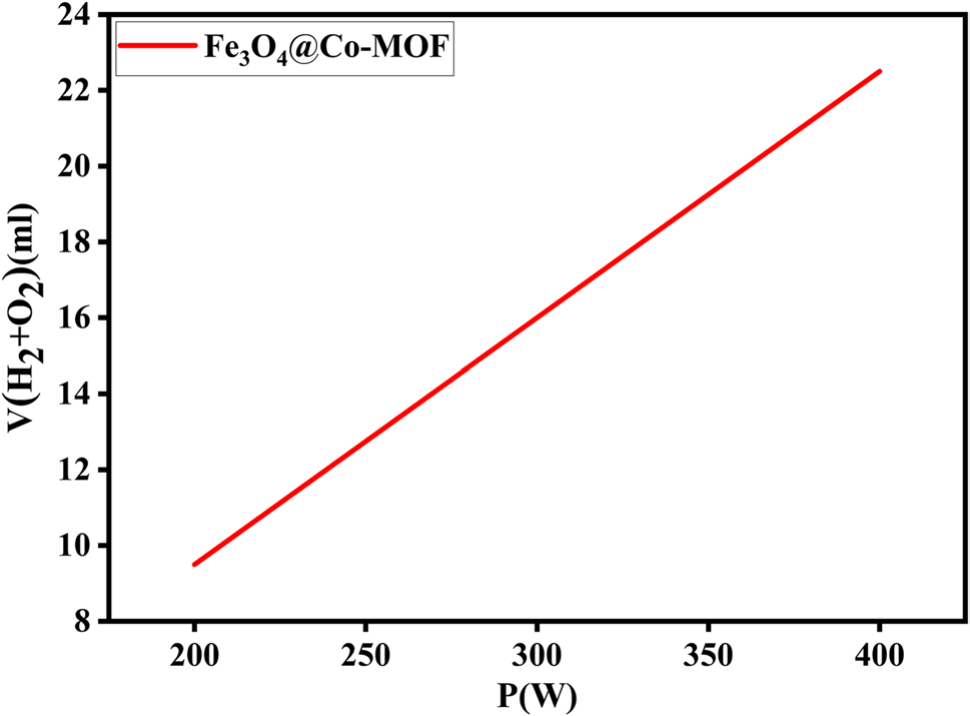
Effects of lamp power

Effect of illumination intensity: The effect of illumination on the catalytic activity of magnetic Co-MOF was investigated as seen in Fig. 11. All experimental conditions were kept constant while the experiment was conducted in the dark (without illumination). The absence of light at ambient temperature led to virtually no hydrogen evolution. A variation in the illumination intensity of the xenon lamp (200 W and 400 W) affected the yield of hydrogen. In the presence of 200 W, minimal hydrogen is generated, but the hydrogen yield increases as illumination intensity increases. Figure 11 illustrates that lamp power has a significant impact on hydrogen generation rates, with high power lamps providing higher yields. The number of incident photons increases along with the intensity of illumination. The result of this is an increase in electrons in the conduction band, which increases hydrogen production.

**Fig. 12:**
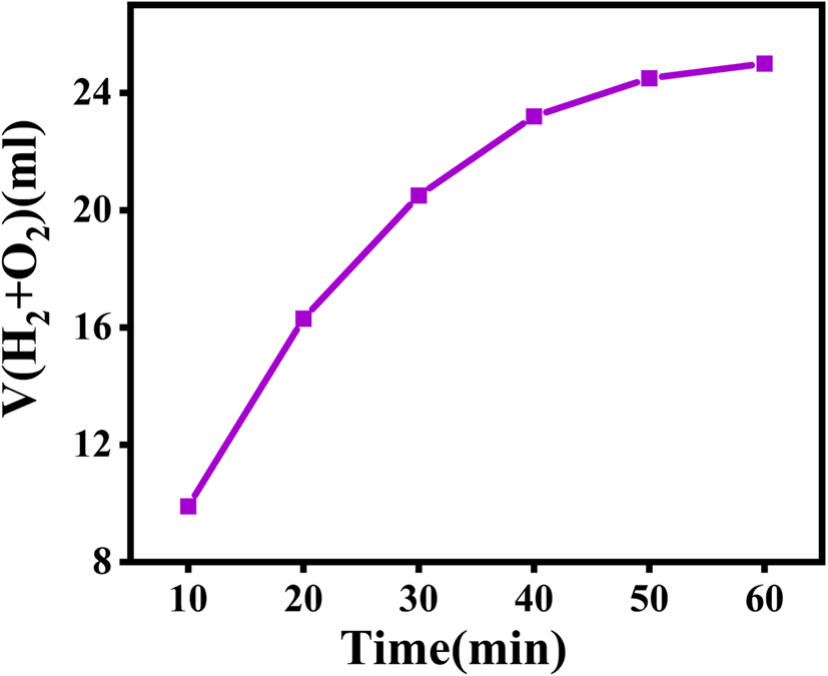
Effects of time

Effect of irradiation time: The effects of varying the irradiation time on photocatalytic hydrogen evolution were studied. In the Fig. 12, it can be seen that the hydrogen evolution rate decreases with increasing irradiation time. Screening of a water splitting reaction for 1 h indicated that the maximum TOF occurred within the first 10 min, after which the gas generated decreased rapidly with a TOF 0.222 of one hour. There is a decrease in hydrogen evolution that can be attributed to backward reactions and a build-up of pressure in the gas phase.

**Fig. 13:**
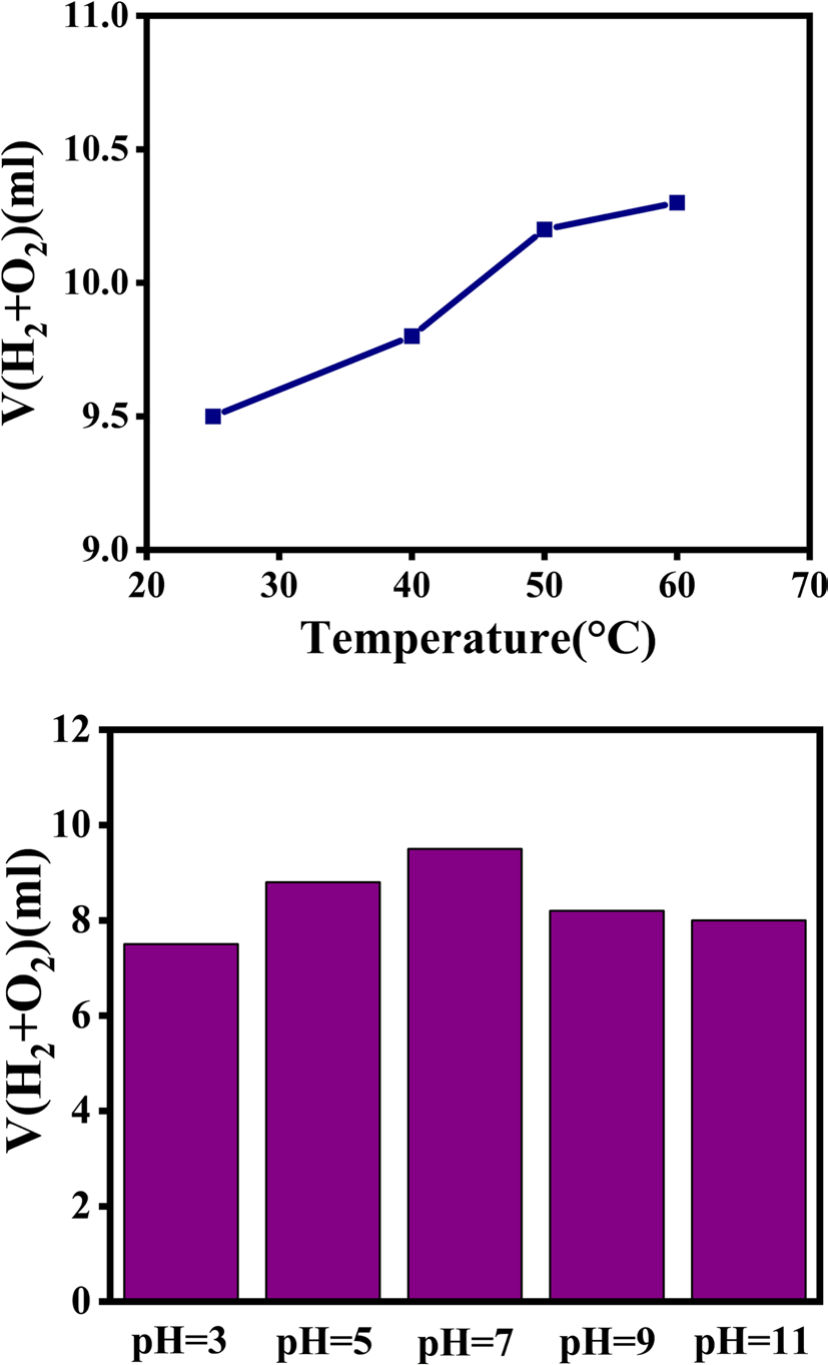
Effects of temperature, and pH

The effect of reaction temperature and pH: optimum temperature and pH were investigated as factors affecting the photocatalytic activity of magnetic metal organic frameworks. As the temperature is raised, the hydrogen evolution rate is observed to increase. Due to the increasing activity of surface sites at higher temperatures, a remarkable effect of temperature was observed on the volume of generated gas. In contrast to the amount of gas produced in acidic and basic media, the impact of pH on the reaction yield was greater at pH=7 (Fig. 13a, b). As acidic media facilitate the evolution of hydrogen, whereas basic media facilitate oxygen evolution, the volume of gas generated in acidic media is higher than that in basic media.

**Fig. 14:**
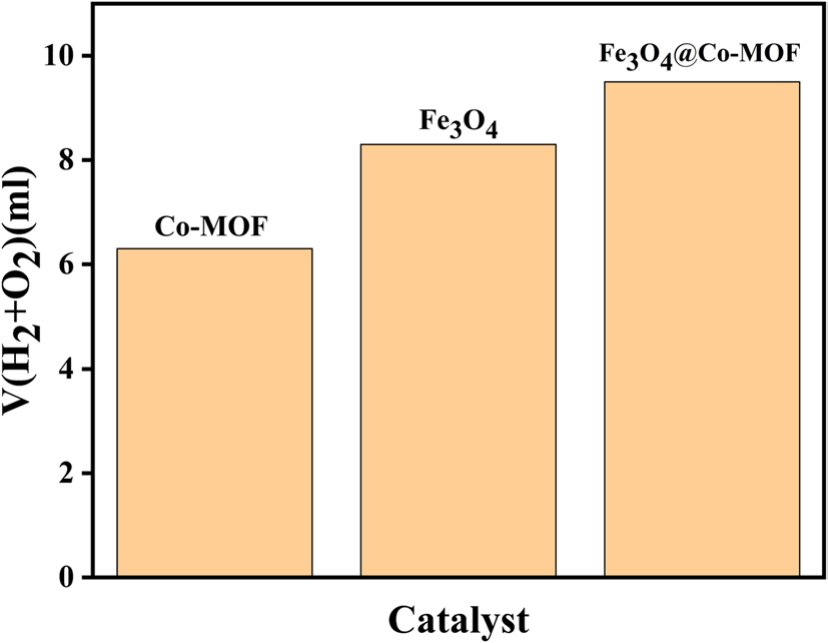
Effects of catalysts

To reveal the effect of Fe_3_O_4_ on the reaction yield, this reaction was done by Co-MOF and Fe_3_O_4_, separately (Fig. 14). The results show that there is a synergic effect in reaction results and the volume of hydrogen gas produced when using Fe_3_O_4_ nanoparticles together with Co-MOF, which can be attributed to the band gap alteration resulted by the addition of Fe_3_O_4_ nanoparticle.

**Fig. 15:**
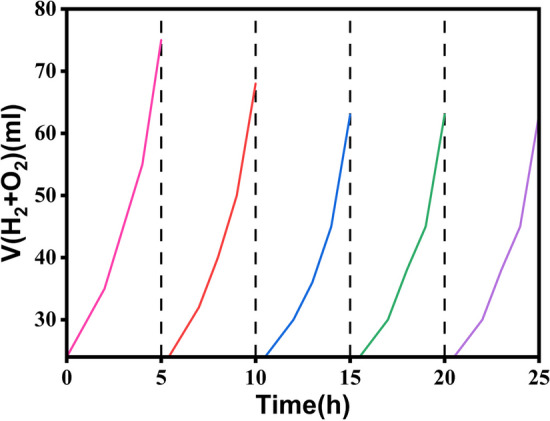
Fe_3_O_4_@Co-MOF system stability testing

The cycle stability of the photocatalyst is very important for its application. Figure 15 shows the stability cycle test of Fe_3_O_4_@Co-MOF at pH = 7, to investigate the performance stability of the Fe_3_O_4_@Co-MOF photocatalyst, recycle experiments were performed and every cycle was held for five h. It is clear that the Fe_3_O_4_@Co-MOF photocatalyst had a slight photo-corrosion which decreased photocatalytic performance during repeated photocatalytic reactions; however, after three cycles of the photocatalytic tests, it is interesting to find that the Fe_3_O_4_@Co-MOF photocatalyst shows a comparable rate constant for the following repeated experiment, suggesting the formation of a stable photocatalytic performance of the photocatalyst.

Due to the intrinsic properties of nanocomposite materials and the ease of recovering the magnetic catalyst in harvesting light, Fe_3_O_4_@Co-MOFs have been shown to be a powerful catalyst for hydrogen evolution from water. As a result of the large surface area for adsorption, the interaction between reactants and photocatalytic materials is greatly enhanced. In addition, the formation of heterojunctions enhances charge carrier separation across the interface, thereby reducing the rate of recombination. With the fabrication of Nano composites, harvesting light capabilities can be extended into the visible or near-IR range. As a final point, the π–π interactions between the major aromatic rings of the organic ligands in MOFs and the surface electrostatic interactions with guest materials will be very helpful in the construction of heterogenous catalysts as well as in facilitating the effective transfer of electrons generated by photons. This work evaluated the photocatalytic activity of Fe_3_O_4_@Co-MOF in the production of hydrogen from water with VIS irradiation at ambient temperature. Under visible light, Fe_3_O_4_@Co-MOF exhibited high activity in the degradation reaction toward H_2_ generation with using 0.01gr of catalyst. A turnover frequency (TOF) of about 0.222 1/h is calculated for the reaction between Fe_3_O_4_@Co-MOF, which is a high value considering the mild reaction conditions. Photocatalytic water splitting has been conducted by various catalysts based on MOF and Fe_3_O_4_ nanoparticles, some of which were selected to be compared with Fe_3_O_4_@Co-MOF considering significant factors including reaction conditions, and TOF (Table 2). However, Fe_3_O_4_@Co-MOF has some advantages, scribed to the mild reaction conditions, highly porous structures, high design flexibility, and easy separation. While Fe_3_O_4_@Co-MOF afforded better yield than some of the previous reports, the most significant benefit of Fe_3_O_4_@Co-MOF is promoting the trend in mild reaction conditions. Finally, the nontoxic nature of the Fe_3_O_4_@Co-MOF makes it a green catalyst compared to the previous ones.

**Table 2:** Comparison of catalysts’ activities in the degradation reaction

Catalyst	TOF (h^−1^)	Refs.
Fe_3_O_4_@MnO_2_	1061.3	[37]
NiPt@MIL-101	65.2	[38]
Ti-MOFs (NH_2_-MIL-125)	40.3	[39]
Pd/TiO_2_/(NH_2_-MIL-125(Ti))	40.3	[39]
In-MOF	777.65	[40]
Mo_2_S_12_@MIL-101(Al)	31.25	[41]
Ni/MIL-101	3238	[42]
Ti-MOF/COF	227	[43]

## Conclusion

We have successfully prepared a magnetic Fe_3_O_4_@Co-MOF composite that exhibits high efficiency in photocatalytic hydrogen production by a simple hydrothermal synthesis method. In this study, the composite showed superior photo-absorption in a wide range of UV and visible wavelengths, making it suitable for use as a photocatalytic material. In the absence of any additives, this photocatalyst promoted water-splitting at ambient temperature with a TOF of 0.222 h^−1^. The presence of mesoporous structures can result in more active sites able to adsorb more substrate water molecules and thereby enhance the efficiency of the photogenerated carriers. Compared to the previous research, the Co-MOF magnetic catalyst is a valuable photocatalytic water splitting catalyst for hydrogen production for the following reasons: (1) High efficiency: the Co-MOF is highly effective at converting solar energy into chemical energy, resulting in higher hydrogen production. (2) stability: As a result of its stability under various reaction conditions and long life, the Co-MOF catalyst is an excellent catalyst for large-scale hydrogen generation while increasing electron transfer rates. (3) low cost: Co-MOF catalysts are cost-effective alternatives compared to other catalysts due to their inexpensive and abundant precursors. (4) tunable properties: these allow the catalyst to be optimized for a variety of reactions. Furthermore, the magnetic properties of the catalyst facilitate easy separation and recovery from reaction mixtures.

## Data Availability

All data related to all experiments are available upon request.

## References

[1] Fan Z, Weng W, Zhou J, Gu D, Xiao W (2021). Progress in research on photocatalytic conversion of carbon dioxide to methanol. J Energy Chem.

[2] Mangrulkar PA, Polshettiwar V, Labhsetwar NK, Varma RS, Rayalu SS (2012). Synthesis of TiO_2_ nanoparticles immobilized on bentonite and its photocatalytic activity in aqueous medium. Nanoscale.

[3] Abas N, Kalair E, Kalair A, ul Hasan Q, Khan N (2020). Hydrogen production methods: current trends and future development prospects. Int J Hydrog Energy.

[4] Hafizi A, Rahimpour M, Hassanajili S (2016). The effect of support surface properties and photocatalytic activity on photoreactor performance. Appl Energy.

[5] Di J, Chen C, Zhu C, Ji M, Xia J, Yan C, Hao W, Li S, Li H, Liu Z (2018). Photocatalytic oxidative degradation of organic pollutants using a nanocomposite catalyst. Appl Catal B Environ.

[6] Kaviyarasu K, Magdalane CM, Jayakumar D, Samson Y, Bashir A, Maaza M, Letsholathebe D, Mahmoud AH, Kennedy J (2020). Enhanced photocatalytic and bactericidal properties of nanostructured materials. J King Saud Univ-Sci.

[7] Qiao Z, Wang W, Liu N, Huang H-T, Karuppasamy L, Yang H-J, Liu C-H, Wu JJ (2022). Recent advances in photocatalytic water splitting using metal sulfide materials. Int J Hydrog Energy.

[8] Xu Y, Zhao W, Xu R, Shi Y, Zhang B (2013). Highly efficient photocatalysts for hydrogen production. Chem Commun.

[9] Ge M, Li Q, Cao C, Huang J, Li S, Zhang S, Chen Z, Zhang K, Al-Deyab SS, Lai Y (2017). Recent advances in the development of efficient photocatalysts. Adv Sci.

[10] Ng YH, Iwase A, Kudo A, Amal R (2010). Solar hydrogen production using semiconductor photocatalysts. J Phys Chem Lett.

[11] Kato H, Asakura K, Kudo A (2003). Photocatalytic water splitting on TiO_2_ materials. J Am Chem Soc.

[12] Guo Y, Feng C, Qiao S, Wang S, Chen T, Zhang L, Zhao Y, Wang J (2020). Enhancing photocatalytic activity with advanced materials. Nanoscale.

[13] Keshipour S, Mohammad-Alizadeh S, Razeghi MH (2022). Improving photocatalytic efficiency using novel materials. J Phys Chem Solids.

[14] Wang R, Gu L, Zhou J, Liu X, Teng F, Li C, Shen Y, Yuan Y (2015). Advanced materials for photocatalytic applications. Adv Mater Interfaces.

[15] Gao S, Cen W, Li Q, Li J, Lu Y, Wang H, Wu Z (2018). Improving the performance of photocatalysts. Appl Catal B Environ.

[16] Sun X, Yu Q, Zhang F, Wei J, Yang P (2016). Recent advances in photocatalytic water splitting. Catal Sci Technol.

[17] Gascon J, Corma A, Kapteijn F, Llabres i Xamena FX (2014). Metal-organic frameworks for photocatalysis. Acs Catal.

[18] He J, Wang J, Chen Y, Zhang J, Duan D, Wang Y, Yan Z (2014). Recent trends in photocatalysis. Chem Commun.

[19] Liu X, Lv X, Lai H, Peng G, Yi Z, Li J (2020). Photocatalytic activity of novel materials. Photochem Photobiol.

[20] Ouyang Y-S, Yang Q-Y (2023). Recent advances in photocatalysis. J Colloid Interface Sci.

[21] Eyvari-Ashnak F, Keshipour S (2023). Molecular catalysis. Mol Catal.

[22] Li T, Jin Z (2022). Advances in photocatalytic water splitting. J Colloid Interface Sci.

[23] Guo J, Liang Y, Liu L, Hu J, Wang H, An W, Cui W (2020). Recent trends in applied surface science. Appl Surf Sci.

[24] Li H, Gong H, Jin Z (2022). Photocatalysis for environmental remediation. Appl Catal B Environ.

[25] Aghayi-Anaraki M, Safarifard V (2020). Recent developments in inorganic chemistry. Eur J Inorgan Chem.

[26] Xiao JD, Jiang HL (2017). Metal-organic frameworks for catalysis. Small.

[27] Kowacs T, O’Reilly L, Pan Q, Huijser A, Lang P, Rau S, Browne WR, Pryce MT, Vos JG (2016). Recent advances in inorganic chemistry. Inorgan Chem.

[28] Ardagh MA, Abdelrahman OA, Dauenhauer PJ (2019). Advances in ACS catalysis. Acs Catal.

[29] Kumaravel V, Imam MD, Badreldin A, Chava RK, Do JY, Kang M, Abdel-Wahab A (2019). Recent trends in photocatalysis. Catalysts.

[30] Li R, Li C (2017). Photocatalytic water splitting on semiconductor-based photocatalysts. Adv Catal.

[31] Yang J, Ma Z, Gao W, Wei M (2017). Recent advances in chemistry. Chem A Eur J.

[32] Meng F, Fang Z, Li Z, Xu W, Wang M, Liu Y, Zhang J, Wang W, Zhao D, Guo X (2013). Recent advances in materials chemistry. J Mater Chem A.

[33] Díaz-García M, Mayoral A, Diaz I, Sanchez-Sanchez M (2014). Crystal growth and design. Cryst Growth Des.

[34] Radhika M, Gopalakrishna B, Chaitra K, Bhatta LKG, Venkatesh K, Kamath MS, Kathyayini N (2020). Materials research express. Mater Res Express.

[35] Gholipoor O, Hosseini SA (2021). New journal of chemistry. New J Chem.

[36] Lee DY, Shinde DV, Yoon SJ, Cho KN, Lee W, Shrestha NK, Han S-H (2014). Recent advances in physical chemistry. J Phys Chem C.

[37] Ameen F, Aygun A, Seyrankaya A, Tiri RNE, Gulbagca F, Kaynak İ, Majrashi N, Orfali R, Dragoi EN, Sen F (2023). Recent advances in environmental research. Environ Res.

[38] Cao N, Yang L, Dai H, Liu T, Su J, Wu X, Luo W, Cheng G (2014). Advances in inorganic chemistry. Inorgan Chem.

[39] Yan B, Zhang L, Tang Z, Al-Mamun M, Zhao H, Su X (2017). Photocatalytic materials for environmental applications. Appl Catal B Environ.

[40] Lu C, Xiong D, Chen C, Wang J, Kong Y, Liu T, Ying S, Yi F-Y (2022). Inorganic chemistry and materials science. Inorgan Chem.

[41] Li H, Yao S, Wu H-L, Qu J-Y, Zhang Z-M, Lu T-B, Lin W, Wang E-B (2018). Photocatalytic applications for environmental protection. Appl Catal B Environ.

[42] Wen M, Cui Y, Kuwahara Y, Mori K, Yamashita H (2016). Photocatalytic materials for energy and environmental applications. ACS Appl Mater Interfaces.

[43] Chen CX, Xiong YY, Zhong X, Lan PC, Wei ZW, Pan H, Su PY, Song Y, Chen YF, Nafady A (2022). Recent advances in Angewandte Chemie. Angew Chem Int Ed.

